# A Rapid Electronic Cognitive Assessment Measure for Multiple Sclerosis: Validation of Cognitive Reaction, an Electronic Version of the Symbol Digit Modalities Test

**DOI:** 10.2196/18234

**Published:** 2020-09-23

**Authors:** Rod M Middleton, Owen R Pearson, Gillian Ingram, Elaine M Craig, William J Rodgers, Hannah Downing-Wood, Joseph Hill, Katherine Tuite-Dalton, Christopher Roberts, Lynne Watson, David V Ford, Richard Nicholas, UK MS Register Research Group

**Affiliations:** 1 Population Data Science Swansea University Medical School Swansea University Swansea United Kingdom; 2 Department of Neurology Morriston Hospital Swansea Bay National Health Service Trust Swansea United Kingdom; 3 Department of Neurology Charing Cross Hospital Imperial College London London United Kingdom

**Keywords:** cognition, multiple sclerosis, eHealth, electronic assessment, patient reported outcomes, neurology

## Abstract

**Background:**

Incorporating cognitive testing into routine clinical practice is a challenge in multiple sclerosis (MS), given the wide spectrum of both cognitive and physical impairments people can have and the time that testing requires. Shortened paper and verbal assessments predominate but still are not used routinely. Computer-based tests are becoming more widespread; however, changes in how a paper test is implemented can impact what exactly is being assessed in an individual. The Symbol Digit Modalities Test (SDMT) is one validated test that forms part of the cognitive batteries used in MS and has some computer-based versions. We developed a tablet-based SDMT variant that has the potential to be ultimately deployed to patients’ own devices.

**Objective:**

This paper aims to develop, validate, and deploy a computer-based SDMT variant, the Cognition Reaction (CoRe) test, that can reliably replicate the characteristics of the paper-based SDMT.

**Methods:**

We carried out analysis using Pearson and intraclass correlations, as well as a Bland-Altman comparison, to examine consistency between the SDMT and CoRe tests and for test-retest reliability. The SDMT and CoRe tests were evaluated for sensitivity to disability levels and age. A novel metric in CoRe was found: question answering velocity could be calculated. This was evaluated in relation to disability levels and age for people with MS and compared with a group of healthy control volunteers.

**Results:**

SDMT and CoRe test scores were highly correlated and consistent with 1-month retest values. Lower scores were seen in patients with higher age and some effect was seen with increasing disability. There was no learning effect evident. Question answering velocity demonstrated a small increase in speed over the 90-second duration of the test in people with MS and healthy controls.

**Conclusions:**

This study validates a computer-based alternative to the SDMT that can be used in clinics and beyond. It enables accurate recording of elements of cognition relevant in MS but offers additional metrics that may offer further value to clinicians and people with MS.

## Introduction

### Background

Multiple sclerosis (MS) is an inflammatory demyelinating and degenerative disease of the central nervous system and the most common nontraumatic cause of disability in young adults worldwide [1]. The dominant phenotype is characterized by relapses (attacks) and remissions, known as relapsing-remitting MS (RRMS). In the majority of those affected with RRMS, the condition evolves, within 10 to 15 years, into secondary progressive MS (SPMS). About 15% of people with MS develop primary progressive MS (PPMS), characterized by progressive neurological dysfunction from onset [2].

Motor impairment forms the most overt impact of MS but cognitive impairment affects up to 40% of people with MS, rising to 80% in those with the progressive forms of the disease [3]. It has substantial impact on disability and can, when present in isolation, limit employment prospects [4]. However, in the early stages of MS, formal cognitive testing can show minimal changes in a wide variety of domains [5]. Later, as the disease advances, the picture becomes more coherent, with impairments in speed of information processing, attention, episodic memory, and executive function dominating. These impact independence and mood and can lead to social isolation [6].

Cognitive testing itself can be demanding on patients, causing difficulties for those with attentional disorders, fatigue, and physical limitations [7]. The time and attention required in a busy clinic environment makes test delivery in a routine context a challenge for both patient and assessors. To this end, in MS, a number of simplified tests of cognition have been developed for clinical use. These include the Brief International Cognitive Assessment for MS [8], the Brief Repeatable Battery of Neuropsychological Tests [9], and the Minimal Assessment of Cognitive Function in Multiple Sclerosis [10]. In most cases, these tests are still largely paper- or apparatus-based exercises completed in front of an assessor and take the form of a battery of tests that incorporate multiple testing methodologies.

One common element of the MS testing batteries is the Symbol Digit Modalities Test (SDMT) [11]. It assesses organic cerebral dysfunction and has a proven history as an effective outcome measure in a number of MS trials [10,11] and in other conditions [12]. The SDMT consists of matching symbols against digits within 90 seconds, the result being the total number of correct answers. Participants are given a practice number of attempts and then perform the timed assessment. The implementation of the test typically takes 5 minutes, including instruction and demonstration. The responses can be written or spoken out loud and recorded by the assessor [13].

A number of electronic variants of the SDMT have been developed [14,15], but as yet, they are not used routinely to assess cognitive impairment [16]. Their implementation varies from the original paper test, but the impact of these slight variations is as yet unclear, as impairment in individuals with MS can vary widely with different elements, such as fatigue, which can slow reactions, and physical issues such as ataxia or weakness, which can introduce further variability if a screen or keyboard needs to be manipulated. This is a further challenge if a test is to be administered without an assessor present. However, the computer-based approaches have the potential to offer additional information over the paper-based or oral approaches, as additional metrics can be quantified and these may enhance the information available from the test.

The United Kingdom Multiple Sclerosis Register (UKMSR) was established in 2011 as a means of capturing real-world evidence of living with MS in the United Kingdom. There are comprehensive data on 11,387 people with MS registered on the UKMSR via the internet and more than 13,000 consented clinically via a network of National Health Service (NHS) centers [17]. An online portal facilitates collection of longitudinal patient-reported outcomes (PROs) and real-world evidence of living with MS, but none of the instruments currently capture cognitive function. Given the need to understand in more depth the performance characteristics of electronic testing and the key role of cognitive impairment in MS, we developed an electronic variant of the SDMT that could be implemented rapidly and routinely at clinical centers to address this need. Ultimately, as an electronic register, if this type of testing is validated, then it could be also carried out in the patient’s home, which would also help patients who are unable to physically attend clinics.

### Objectives

This paper aims to develop, validate, and deploy a computer-based SDMT variant, the Cognition Reaction (CoRe) test, that can reliably replicate the characteristics of the paper-based SDMT and assess its utility for deployment as a meaningful measure to assess cognition in an MS population.

## Methods

### Population

All participants gave informed consent, and the study has ethical approval from South West Central Bristol Ethics Committee (16/SW/0194). Participants were recruited from Morriston Hospital, Swansea Bay University Health Board and Charing Cross Hospital, Imperial College Healthcare NHS Trust. The people with MS that took part in the study were recruited at either progressive MS teaching days or as part of their routine visits to their respective hospitals. Demographic data and an Expanded Disability Status Score (EDSS) [18] were recorded at the time of testing. Healthy volunteers were recruited from Swansea University Medical School and Imperial College London to provide a control group of test scores with anonymized demographic data. Healthy volunteers were recruited from among the staff at the two clinical sites and included a mix of staff and PhD students from Swansea University. None of the healthy controls had MS and no one approached refused. All participants had completed at least full formal secondary education. There were no declared visual problems in the population.

### CoRe Test App

**Figure 1:**
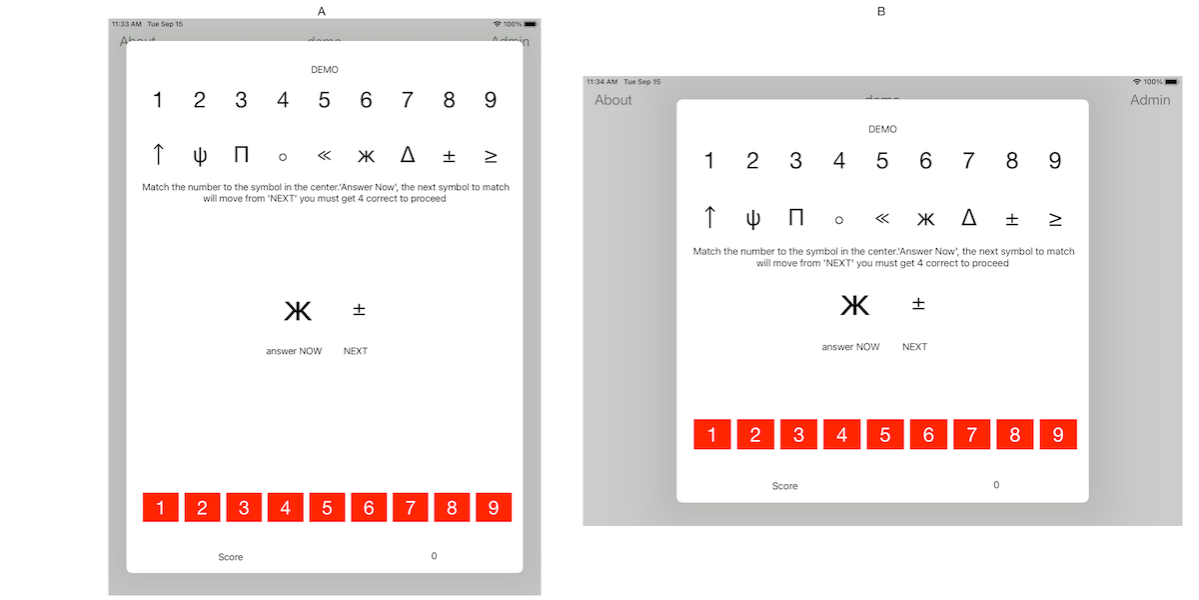
Cognitive Reaction test app shown running in portrait and landscape modes.

The Cognition Reaction (CoRe) test was inspired by the SDMT; however, there are some key differences. The CoRe test presents 9 different symbols displayed at the top of the screen, with corresponding numbers, 1 through 9, underneath. The symbols are randomized every time the app is launched, and the center of the screen displays 2 symbols, the one to be identified now and the next one. At the bottom of the screen, there are a number of buttons labelled 1 through 9 that participants tap to match the central symbol on the screen. Data recorded include the number of symbols accurately tapped, as for the SDMT, but in addition, CoRe automatically registers the time between responses and the number of incorrect responses. Further details of the app are presented in Multimedia Appendix 1 [19,20]. The app is entirely self-contained, with no requirement for internet access. The CoRe test app can be seen in Figure 1.

### App Testing

For the MS population, participants first completed the paper SDMT using the traditional written method, requiring the paper test, a pen, and a stopwatch. Following this, participants were handed an iPad and given an introduction by a researcher from the UKMSR team, merely demonstrating the 2 orientations that the device could be placed in. The orientation that participants chose was not recorded as part of this assessment. They were then invited to follow the written directions on the app. They were first presented with a demonstration mode and encouraged to run through at least once. A score of 4, which was displayed on the screen, was required to progress to the main test. This could be repeated if desired. Once ready, participants hit “start” and were given 90 seconds to complete the test. A countdown timer was displayed on the screen of the iPad. Visual acuity was not formally assessed, and no participants claimed that they could not see the icons on the tablet screen. Test environments were controlled for noise and disturbance. Some participants were retested 1 month later in the same environment to determine the consistency of the results.

### Statistical Analysis

Analysis was carried out using the Pandas library for Python (version 3.773) [21] and the R statistical language (version 3.6.0; R Foundation for Statistical Computing) [22]. Graphs and images were generated using Seaborn [23] and ggplot2 (version 3.0.0) [24]. Correlation was used to compare the validity of the paper and electronic versions of the tests and the test-retest reliability of the CoRe test. Pearson *r* was calculated for test scores from the CoRe test and the SDMT, with mean difference evaluated using a 2-tailed paired samples *t* test and differences in variances compared using a Pitman-Morgan test for paired samples. Intraclass correlation was also performed on the first and second CoRe and SDMT test results. A Bland-Altman analysis was used as an additional measure of equivalency. The sensitivity of the CoRe and SDMT scores to disability levels and age were measured using analysis of variance (ANOVA) statistics, with post hoc Tukey tests used to determine any significant differences between groups.

To utilize the additional data generated by the CoRe test, the question answering velocity (QAV) was quantified as a measure of cognitive function. This was defined as the total number of correct answers given at a time divided by total current time elapsed in the test (correct answers/seconds). Multivariate linear regression was performed to determine if any relationship existed between the QAV and the time period of the questionnaire. The CoRe test lasts a total of 90 seconds, and responses were divided into thirds to study the rates of change over the first, second, and third sections of responses for each patient. For analysis, EDSS scores were divided into 3 categories: low (EDSS of 0-2.5), medium (EDSS of 3-5.5), and high (EDSS of 6-10), as was age, with categories of 18-34 years, 35-54 years, and >55 years.

## Results

### Demographics

A total of 102 people with MS were recruited to the study (Table 1), of whom 30 returned within 1 month for a repeat test. All patients were over 18 years of age and had no significant comorbidities that would exclude them from being able to complete the paper or CoRe tests. No participants were excluded from the study, and none reported a relapse of MS at any point in the testing. Mean age of the people with MS cohort tested was younger than the overall MS Register population, with a slightly lower proportion of patients with PPMS and SPMS (Table 1). A total of 45 anonymous healthy controls were tested during the development of the app; apart from not completing an initial paper SDMT, conditions were similar to the MS cohort. Both healthy controls and people with MS had completed at least 12 years of education.

**Table 1:** Demographics of cohort and healthy controls undertaking the CoRe test. The UKMSR population is shown for comparison.

Characteristic		Total UKMSR^a^	CoRe^b^ cohort (MS^c^)	CoRe cohort (healthy controls)	Cohort difference^d,^ chi-square test (*df*)	Cohort difference^d^, *t* test (*df*)	*P* value
Total participants, n		11,387	102	45	N/A^e^	N/A	N/A
**Gender, n (% )**					0.3 (1)	N/A	.57
	Female	8387 (73.7)	70 (68.6)	28 (62.2)			
	Male	3000 (26.3)	32 (31.4)	17 (37.8)			
**MS type, n (%)**					N/A	N/A	N/A
	RRMS^f^	5988 (52.6)	86 (83.2)	N/A			
	PPMS^g^	1492 (13.1)	5 (5.6)	N/A			
	SPMS^h^	2945 (25.9)	9 (9.3)	N/A			
	Other	962 (8.4)	2 (1.9)	N/A			
Age (years), mean (SD)		53.6 (11.8)	44.0 (11.0)	38.1 (11.9)	N/A	2.891 (145)	.004
Age at diagnosis (years), mean (SD)		39.2 (10.3)	34.6 (10.6)	N/A	N/A	N/A	N/A
EDSS^i^, median (range)		6 (0-9.5)	3.5 (1-8)	N/A	N/A	N/A	N/A

^a^UKMSR: United Kingdom Multiple Sclerosis Register.

^b^CoRe: Cognitive Reaction.

^c^MS: multiple sclerosis.

^d^Difference between people with multiple sclerosis and healthy controls.

^e^N/A: not applicable.

^f^RRMS: relapsing-remitting multiple sclerosis.

^g^PPMS: primary progressive multiple sclerosis.

^h^SPMS: secondary progressive multiple sclerosis.

^i^EDSS: Expanded Disability Status Score.

### CoRe Test in People With MS and Control Group: Comparison of Total Correct Responses

The first set of CoRe test scores for people with MS were compared with those of the healthy control group. Mean test results for people with MS were 39.0 (SD 13.3), while mean scores for the healthy control group were 56.1 (SD 15.9). An unpaired *t* test found that people with MS had significantly lower scores (t_145_=–6.769; *P*<.001), with no significant difference in variance between the groups (*F*_101,44_=0.701; *P*=.15).

### CoRe Test and SDMT in People With MS: Comparison of Total Correct Responses

**Figure 2:**
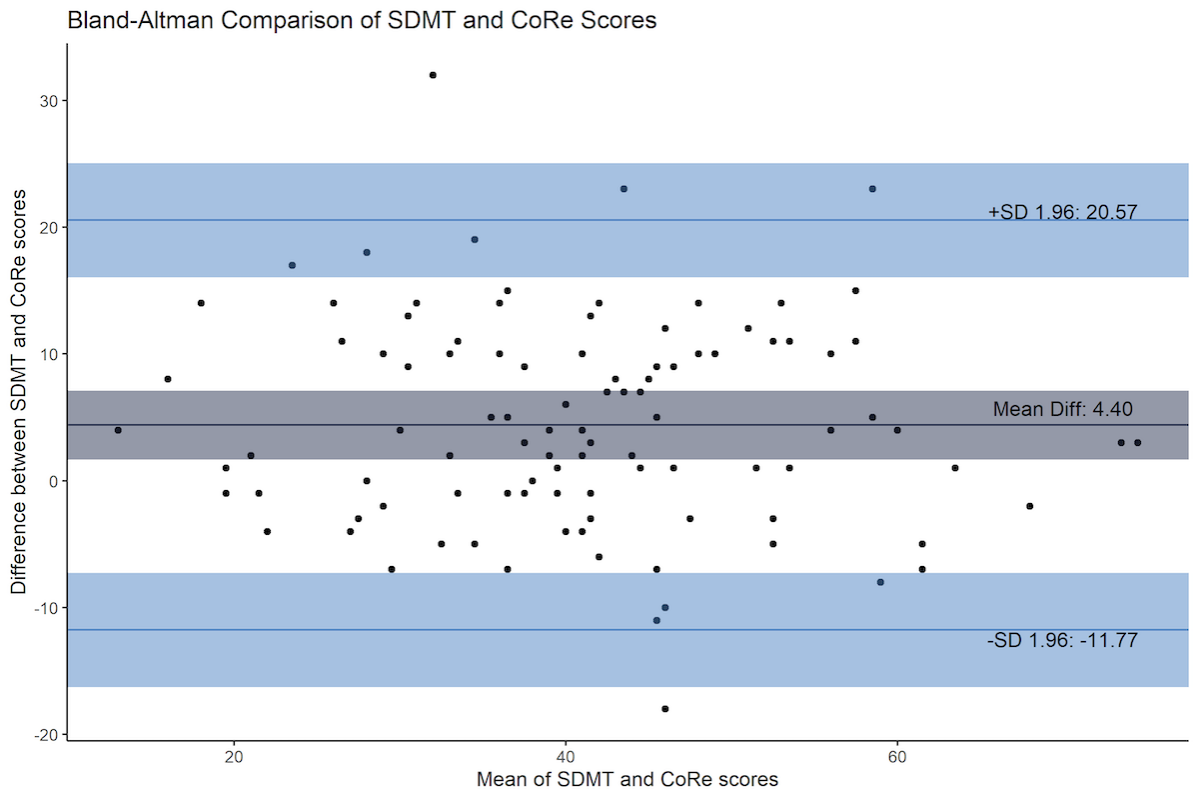
Bland-Altman comparison of first CoRe test with paper SDMT scores. CoRe: Cognitive Reaction; SDMT: Symbol Digit Modalities Test.

People with MS completed both the CoRe test and SDMT together on 2 occasions, 1 month apart. The first test response distributions for the CoRe test and SDMT were normally distributed (Shapiro-Wilk tests with *P*=.48 and *P*=.61, respectively) and were strongly correlated (Pearson *r*_100_=0.800; *P*<.001). First test participants scored a mean of 4.40 responses lower for the CoRe test compared with the SDMT, as seen in Table 2 (paired samples t_101_=5.390; *P*<.001), but there was no significant difference in the variance (Pitman-Morgan test: t_100_=–0.879; *P*=.38), with good agreement between tests (Figure 2). When the CoRe test and SDMT were repeated for a second time, the mean CoRe test score was not significantly lower than the SDMT (1.4 responses difference; t_29_=0.954; *P*=.35). Again, there was a strong correlation between the second CoRe test and second SDMT (Pearson *r*_28_=0.842; *P*<.001). Table 2 shows the baseline and retest responses for those who completed it.

**Table 2:** Baseline and retest SDMT and CoRe test total responses at baseline and retest 1 month later.

Test		Participants, n	Score, mean (SD), range
**Baseline**			
	SDMT^a^	102	43.4 (12.6), 15-76
	CoRe^b^ test	102	39.0 (13.3), 11-73
**Retest**			
	SDMT	30	41.9 (14.6), 14-76
	CoRe test	30	40.5 (13.9), 20-70

^a^SDMT: Symbol Digit Modalities Test.

^b^CoRe: Cognitive Reaction.

### CoRe Test and SDMT Test-Retest Reliability

**Figure 3:**
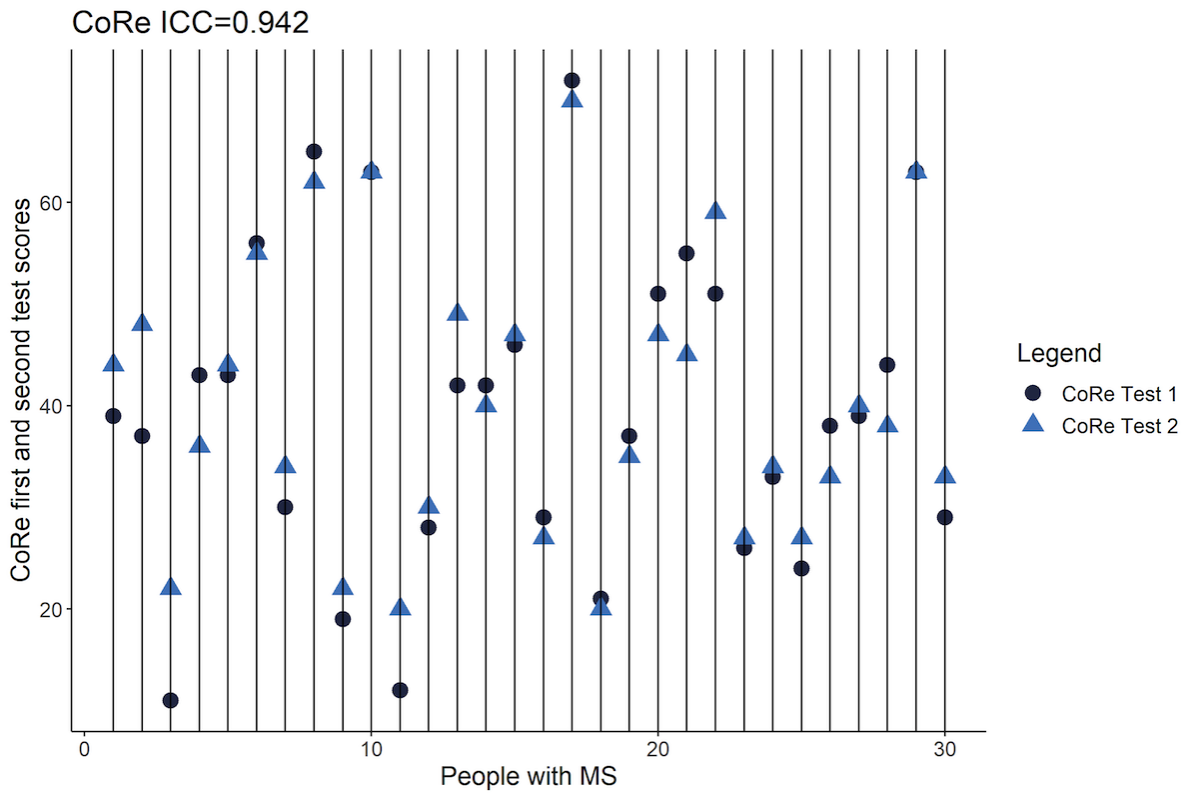
Intraclass correlation coefficients between the first and retested CoRe tests. CoRe: Cognitive Reaction; ICC: intraclass correlation coefficient; MS: multiple sclerosis.

First and second CoRe test and SDMT scores were evaluated for test-retest reliability and scores at a 1-month interval. The CoRe tests were normally distributed and demonstrated consistency (Pearson correlation coefficient *r*=0.947; t_28_=15.60; *P*<.001). Differences in means were normal (Shapiro-Wilk test *P*=.81) and not significantly different (t_29_=–0.944; *P*=.35), with equal variances (Pitman-Morgan t_28_=1.784; *P*=.09). The intraclass correlation coefficient between the first and second CoRe tests was found to be 0.942 (95% CI 0.882-0.0972; *F*_29,30_=33.2; *P*<.001) (Figure 3).

Test-retest correlations were observed in the same people completing the SDMT at a 1-month interval. Scores were normally distributed and consistent (Pearson correlation *r*=0.936; t_28_=14.052; *P*<.001) and differences in means were normal (Shapiro-Wilk test *P*=.44) and not significantly different (t_29_=–0.919; *P*=.37), with equal variances (Pitman-Morgan t_28_=–0.743; *P*=.46). The intraclass correlation coefficient between the first and second SDMT tests was found to be 0.935 (95% CI 0.869-0.968; *F*_29,30_=29.6; *P*<.001).

### CoRe Test Total Correct Response Score Is Impacted by Age and Disability in MS, Whereas SDMT Is Only Affected by Disability

**Figure 4:**
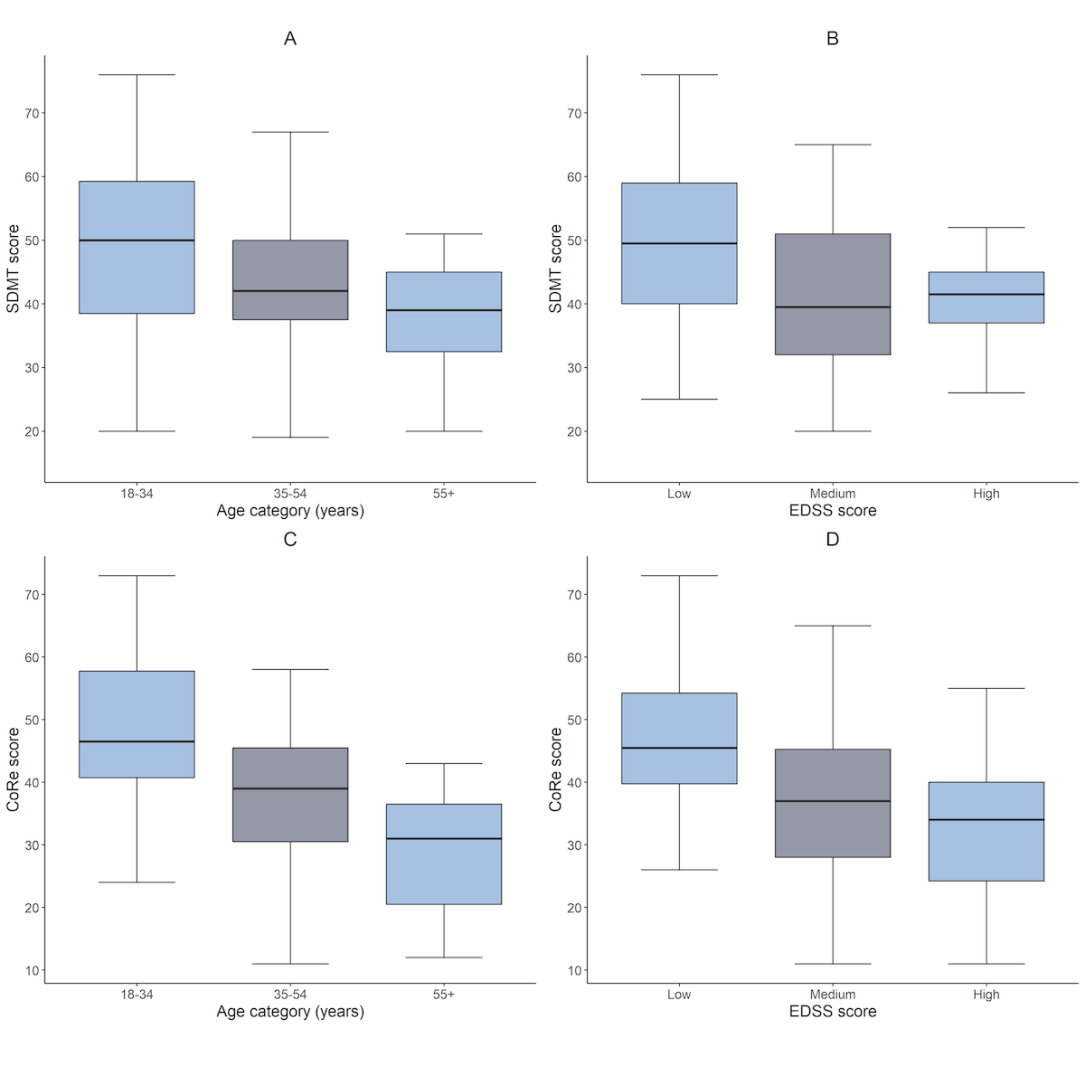
Mean SDMT and CoRe scores with age categories and EDSS scores. CoRe: Cognitive Reaction; EDSS: Expanded Disability Status Score; SDMT: Symbol Digit Modalities Test.

We examined the impact of age, gender, and EDSS on the total correct responses (Figure 4). An ANOVA for SDMT scores with respect to age and EDSS found no significant impact of age (aged 18-34 years: mean 48.1, SD 15.5; aged 35-54 years: mean 43.2, SD 11.8; and 55+ years: mean 38.3, SD 9.0; *F*_2_=1.036; *P*=.36), but significance for EDSS (low EDSS: mean 49.8, SD 12.9; medium EDSS: mean 41.4, SD 12.3; high EDSS: mean 38.6, SD 9.8; *F*_2_=8.574; *P*<.001); post hoc Tukey tests showed higher scores in those in the lowest EDSS category compared with those in the highest EDSS category (*P*<.001) and compared with the medium EDSS category (*P*=.01). No significant difference was found between the low and medium EDSS categories*.*

In contrast, an ANOVA for CoRe test scores showed a significant difference in the total responses with age (aged 18-34 years: mean 48.6, SD 13.5; aged 35-54 years: mean 38.3, SD 11.6; and >55 years: mean 28.9, SD 9.8; *F*_2_=8.633; *P*<.001) and EDSS (low EDSS: mean 47.4, SD 11.6; medium EDSS: mean 36.8, SD 12.7; high EDSS: mean 32.1, SD 10.7; *F*_2_=18.151; *P*<.001). Post hoc Tukey tests showed those in the age group of 18 to 34 years had significantly higher scores than those in the 34 to 54 years (*P*=.01) and 55+ years group (*P*=.001), with no difference between the medium and high age groups. The lowest EDSS category was associated with higher CoRe test scores than both other groups (*P*<.001), with no difference between the medium and high EDSS groups.

Gender was not found to be significant for either SDMT or CoRe test scores.

### Speed of Reaction (Question Answering Velocity) Derived From the CoRe Test Increases Throughout the Test and Correlates With Age, Gender, and Disability

**Figure 5:**
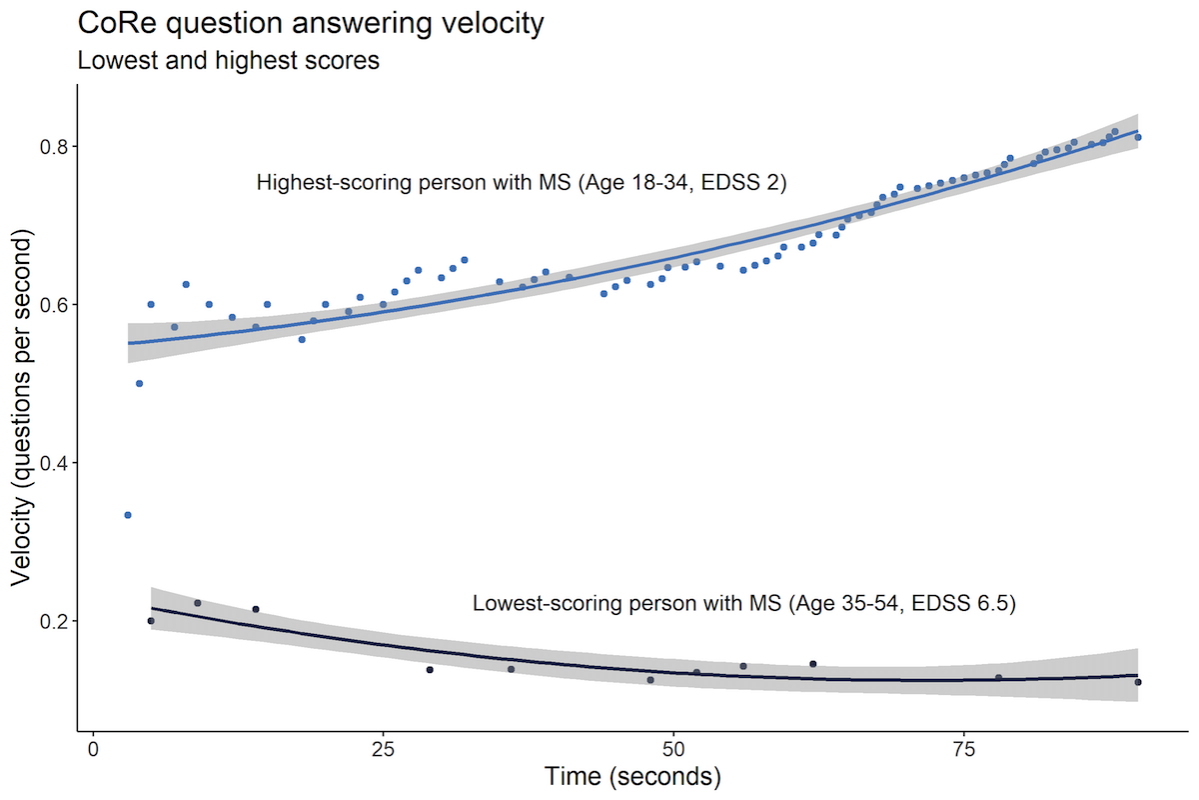
A polynomial regression of QAV for those people with MS with the lowest and highest scores in the cohort. CoRe: Cognitive Reaction; EDSS: Expanded Disability Status Score; MS: multiple sclerosis; QAV: question answering velocity.

Due to the way data are acquired for the CoRe test, we were able to measure the speed of reaction to each question and calculate the QAV as correct answers over time elapsed (seconds) continuously throughout the assessment. There was a significant range of QAV over the time frame of the test in people with MS, as illustrated in Figure 5, which shows the two individuals with the lowest and highest scores in the CoRe test. Breaking down the total correct answers into 3 sections also allowed us to quantify the change in QAV over the course of the CoRe test. Multiple linear regression models with the variables age, gender, and EDSS in people with MS found that QAV increased in each third of the test in people with MS and healthy controls (Table 3).

**Table 3:** Multivariate models in people with MS (R^2^=0.396; F_5,3973_=520.4; *P*<.001) and healthy controls (R^2^=0.323; F_4,2521_=300.1; *P*<.001) for QAV over the time frame of the Cognitive Reaction test, with additional covariates age and gender. EDSS scores are given for people with MS only.

Variable	QAV^a^ of people with MS^b^			QAV of healthy controls	
	β coefficient (95% CI)	*P* value	β coefficient (95% CI)		*P* value
Second section compared to first	.045 (0.037 to 0.053)	<.001	.070 (0.056 to 0.085)		<.001
Third section compared to first	.071 (0.063 to 0.080)	<.001	.110 (0.094 to 0.123)		<.001
Age	–.005 (–0.005 to –0.006)	<.001	–.008 (–0.007 to –0.008)		<.001
Female gender	.049 (0.041 to 0.056)	<.001	–.043 (–0.055 to –0.031)		<.001
EDSS^c^	–.017 (–0.015 to –0.019)	<.001	N/A^d^		N/A

^a^QAV: question answering velocity.

^b^MS: multiple sclerosis.

^c^EDSS: Expanded Disability Status Score.

^d^N/A: not applicable.

**Figure 6:**
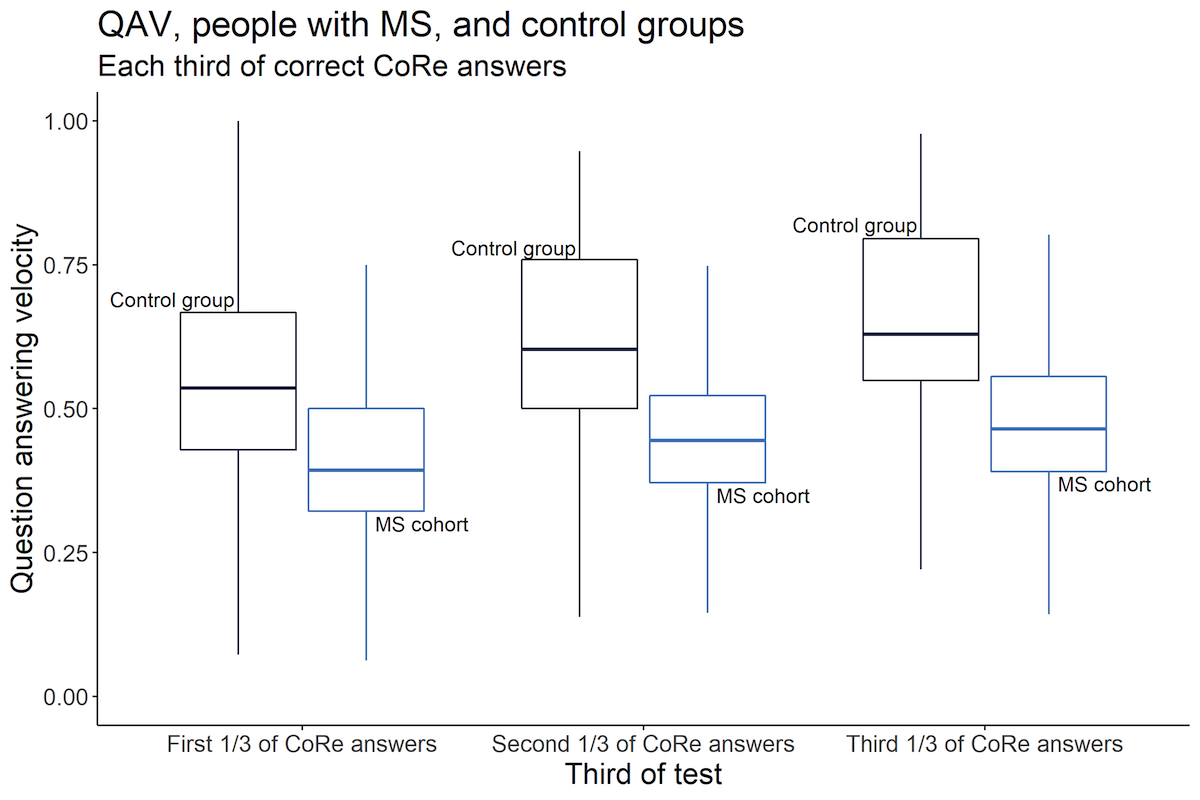
Comparison of increase in speed between each test third for healthy and MS populations. CoRe: Cognitive Reaction; MS: multiple sclerosis; QAV: question answering velocity.

Both groups answered more quickly as the test progressed (the control group at an even faster rate than people with MS), with the second and third sections of their correct answers being completed in less time than the first. The gradient is similar in both populations (Figure 6). In both populations, increased age was associated with slowing of QAV by 0.007 to 0.008 questions per second for each year increase in age. For control participants, female gender was associated with a slowing of QAV by 0.034 questions per second, whereas in people with MS, female gender was associated with an increase in QAV of 0.049 questions per second. However, disability slowed QAV by 0.017 questions per second for every increase in EDSS by 1 point (Table 3).

We next directly compared the variables associated with CoRe test QAV and the CoRe test total response score. A regression using the variables age, gender, and EDSS score found that the CoRe test QAV was significantly impacted by all 3 factors, whereas the CoRe test score (total correct answers) found significant impacts only from EDSS and age (Table 4).

**Table 4:** Impact of age, gender, and EDSS on total response score (R^2^=0.383; F_3,98_=20.3; *P*<.001) in people with MS cohort.

Variable	CoRe^a^ test score	
	β coefficient (95% CI)	*P* value
EDSS^b^	–2.103 (–3.390 to –0.808)	.002
Age	–.489 (–0.713 to –0.265)	<.001
Female gender	4.413 (–0.155 to 8.981)	.06

^a^CoRe: Cognitive Reaction.

^b^EDSS: Expanded Disability Status Score.

## Discussion

### Summary of Findings

This study aims to validate an electronic variant of the SDMT, comparing the CoRe test with the established paper-based SDMT within an MS cohort in 2 independent UK centers, examining its overall reliability and suitability. In addition, we quantified an additional metric that can be extracted from the electronic implementation. The total response scores for the CoRe test were on average lower than the SDMT but showed good correlation with the paper test, though there are clear differences in responses across age groups. Having the understanding that the CoRe performs similarly across these deviations allows it to be compared with the paper-based test, though it is not a like-for-like match. However, the consistency of the test and its utility remain clear. The CoRe test showed consistent responses over time and demonstrated similar test-retest properties to the SDMT, as with other electronic implementations [14]. These findings suggest that the CoRe test is an appropriate alternative to measure of cognitive ability as assessed by the SDMT.

We confirmed that a reduction in correct responses for both the SDMT and CoRe test correlates with increasing disability, but in addition, a reduction in correct responses correlates with increasing age in people with MS. Using the advantages of an electronic implementation, we were able to measure the QAV and found that both people with MS and healthy controls increase their QAV throughout the test and also that in both groups, an increased QAV correlates with younger age and male gender. This implies these correlations are not associated with MS-specific cognitive decline. However, increased QAV is also associated with lower disability, only present in those with MS. In our testing, increasing age showed a reduction in correct responses over the test. This finding corresponds with other SDMT testing in populations [25], and there is evidence for older participants performing poorly over the duration of the test, with studies showing decreased reaction times (about 0.5 ms/year) [26] in simple reaction-style tests in older people. There is also the effect of older people’s familiarity with tablet computers [27] that could have some impact on this. This will be investigated in future testing.

There are a number of computer-based variants of the SDMT, one of the first being the computerized Symbol Digit Modalities Test (c-SDMT) [14], which showed excellent sensitivity in 119 people with MS versus 38 healthy controls, with people with MS performing significantly worse than the healthy controls. Use of the c-SDMT has not become widespread, most likely due to the technology platform that it was developed on and the stringent test description (Windows PC, 19-inch screen with participant at 15 inches from the screen), making deployment challenging. A more recent implementation of a computer-based SDMT is the processing speed test (PST) [15], which was also tested against a healthy control population and forms one element of the Floodlight assessment tool [28]. The PST showed similar results as we have demonstrated and has shown high reliability when reproduced within Floodlight on patients’ own devices. Small differences in implementation of the same paper-based test can impact what is being tested and need to be understood. The CoRe implementation requires the screen to be touched, which adds a visuospatial element to the assessment, and this will have an impact in some people with MS. It also presents 2 symbols in random order as opposed to a standard sheet of symbols; this change means that there is less likely to be a learning effect on retesting. A key issue with computer-based implementation is the impact of rapid hardware and software development, which results in a need to develop applications that can adapt to a changing environment. Another issue is the variety of devices, such as desktops, laptops, and smartphones, that are currently in use, especially if the test is to be performed without an assessor present. CoRe has been developed to run at multiple screen sizes and on different devices, with an interface—two symbols seen at a time—that is suited to a small screen. This will have to be tested separately.

Prior studies, and our results, show that data produced by electronic tests are consistent, repeatable, and have utility to clinicians, informing on a vital aspect of patient care [29]. The scores on both paper SDMT and the CoRe test fall with increasing disability. The CoRe test is more sensitive than the SDMT to age, with the SDMT being only affected in populations older than 55 years [27]. The electronic CoRe test allows greater analysis of this effect, demonstrating slower mean response times in higher ages and disability groups. There is some evidence that there may be a gender difference in cognitive tests [30], with males and females performing differently at various ages in different test types. Notably, this is seen with visual reaction times, and this would be consistent with the implementation of the test presented here. The fact that this extra variable of reaction time (QAV) can be measured as part of the CoRe test could have clinical or research utility in the future. Having additional quantifiable clinical measurement information via a simple-to-implement and rapid test could hopefully have some relevance to everyday clinical practice, research, and medication trials. Benedict et al [13] state that the current definition of “NEDA” (no evidence of disease activity) is predicated on largely physical outcome measures, but cognition is so fundamental to socialization, employment, and quality of life beyond pure health care that a prolonged measurement of cognitive aspects could add a compelling dimension to our understanding of disease activity.

### Limitations

We identified some limitations with this study. First, there were few people with MS with progressive disease and advanced disability, and we did not have complete directly measured cognitive assessments. In addition, the population that took 1-month follow-up tests was limited, and we have only tested this on a single type of device here. The 1-month period chosen for retest represents the hospital visit pattern for some patients on a particular disease-modifying therapy. Differing retest periods should be tested in the future. Although testing was performed in the presence of a researcher, they had little or no input on the actual test itself—though this has been shown to not have effect on these types of tests [31]. We also did not consider the orientation of the device as having any effect. This could also be incorporated into future testing on other devices.

Given that the CoRe test is consistent and repeatable, we intend to test the app on other devices, including laptops and a variety of smartphones. This will facilitate completion of the test away from the clinic and will enable us to integrate the CoRe test into the range of PROs captured by the UKMSR. Additionally, this will allow us to carry out testing among participants with higher disability and more progressive disease at different intervals to ensure that the test maintains its reliability and repeatability. We recognize that the CoRe is not an exact replacement for SDMT. It is an entirely new test [32], but it is comparable and measurable compared with the SDMT.

### Conclusion

The CoRe implementation of the SDMT test is reliable and correlates with the paper-based SDMT, while also offering the additional metric of patient reaction time (QAV). This will allow clinicians and researchers to capture important additional metrics in people with MS, and potentially in other diseases, quickly and reliably on existing tablet hardware.

## Appendix

Multimedia Appendix 1 CoRe test Application Details.

## Abbreviations

ANOVA: analysis of variance
CoRe: Cognitive Reaction
c-SDMT: computerized Symbol Digit Modalities Test
EDSS: Expanded Disability Status Score
MS: multiple sclerosis
NHS: National Health Service
PPMS: primary progressive multiple sclerosis
PRO: patient-reported outcome
PST: processing speed test
QAV: question answering velocity
RRMS: relapsing-remitting multiple sclerosis
SDMT: Symbol Digit Modalities Test
SPMS: secondary progressive multiple sclerosis
UKMSR: United Kingdom Multiple Sclerosis Register
