# Supplementary material for: A Rapid Electronic Cognitive Assessment Measure for Multiple Sclerosis: Validation of Cognitive Reaction, an Electronic Version of the Symbol Digit Modalities Test
Source: J Med Internet Res. 2020 Sep 23;22(9):e18234. doi: 10.2196/18234 (PMC7542403; doi:10.2196/18234)
Supplement: Multimedia Appendix 1 [file jmir_v22i9e18234_app1.docx]

**Appendix 1: CoRe test Application Details**

The Cognitive Reaction test (CoRe test) was designed and built to work on all Apple iPad devices running on iOS 11 and above. The application was written in the Swift Language (version 4) [[31]] and developed using Apple Xcode 10 [[32]]. The application was deployed with the ‘Testflight’ application so that the CoRe test could be consistently deployed to enrolled and controlled iPads. No data is streamed to or from the internet at any point.

The CoRe test can be administered in either portrait or landscape mode. As the predefined set of 9 potential glyphs is randomised every time the application is launched, and as each symbol may only appear once in that iteration, the CoRe test provides a potential of 362,880 unique combinations. Showing the current and upcoming symbol allows pre-processing and mimics the SDMT (where matches still to be completed can all be seen on the paper). The size and appearance of the glyphs do not change. Only the order of their appearance *between* tests changes.

The basic engine of the application has been optimised for a number of screen and device types, and will work – but has not been tested on iPhones and smaller tablets.
